# Clinical framework for next generation sequencing based analysis of treatment predictive mutations and multiplexed gene fusion detection in non-small cell lung cancer

**DOI:** 10.18632/oncotarget.16276

**Published:** 2017-03-16

**Authors:** Kajsa Ericson Lindquist, Anna Karlsson, Per Levéen, Hans Brunnström, Christel Reuterswärd, Karolina Holm, Mats Jönsson, Karin Annersten, Frida Rosengren, Karin Jirström, Jaroslaw Kosieradzki, Lars Ek, Åke Borg, Maria Planck, Göran Jönsson, Johan Staaf

**Affiliations:** ^1^ Department of Pathology, Regional Laboratories Region Skåne, Lund SE 22185, Sweden; ^2^ Division of Oncology and Pathology, Department of Clinical Sciences Lund, Lund University, Medicon Village, Lund SE 22381, Sweden; ^3^ Division of Oncology and Pathology, Department of Clinical Sciences Lund, Lund University, Lund SE 22185, Sweden; ^4^ Department of Respiratory Medicine and Allergology, Skane University Hospital, Lund SE22185, Sweden; ^5^ CREATE Health Strategic Center for Translational Cancer Research, Lund University, Medicon Village, Lund SE 22381, Sweden; ^6^ Department of Oncology, Skåne University Hospital, Lund SE 22381, Sweden

**Keywords:** lung cancer, NGS, gene fusion, mutation, precision medicine

## Abstract

Precision medicine requires accurate multi-gene clinical diagnostics. We describe the implementation of an Illumina TruSight Tumor (TST) clinical NGS diagnostic framework and parallel validation of a NanoString RNA-based *ALK*, *RET*, and *ROS1* gene fusion assay for combined analysis of treatment predictive alterations in non-small cell lung cancer (NSCLC) in a regional healthcare region of Sweden (Scandinavia). The TST panel was clinically validated in 81 tumors (99% hotspot mutation concordance), after which 533 consecutive NSCLCs were collected during one-year of routine clinical analysis in the healthcare region (˜90% advanced stage patients). The NanoString assay was evaluated in 169 of 533 cases. In the 533-sample cohort 79% had 1-2 variants, 12% >2 variants and 9% no detected variants. Ten gene fusions (five *ALK*, three *RET*, two *ROS1*) were detected in 135 successfully analyzed cases (80% analysis success rate). No *ALK* or *ROS1* FISH fusion positive case was missed by the NanoString assay. Stratification of the 533-sample cohort based on actionable alterations in 11 oncogenes revealed that 66% of adenocarcinomas, 13% of squamous carcinoma (SqCC) and 56% of NSCLC not otherwise specified harbored ≥1 alteration. In adenocarcinoma, 10.6% of patients (50.3% if including *KRAS*) could potentially be eligible for emerging therapeutics, in addition to the 15.3% of patients eligible for standard EGFR or ALK inhibitors. For squamous carcinoma corresponding proportions were 4.4% (11.1% with *KRAS*) vs 2.2%. In conclusion, multiplexed NGS and gene fusion analyses are feasible in NSCLC for clinical diagnostics, identifying notable proportions of patients potentially eligible for emerging molecular therapeutics.

## INTRODUCTION

Discoveries of frequent and therapeutically targetable mutations and gene fusions in non-small cell lung cancer (NSCLC) have changed not only the clinical management of the disease, but also the procedures and techniques used in the diagnosis of the disease. In addition to the current cornerstones of targeted therapy in NSCLC, *EGFR* mutations and *ALK* gene fusions, a growing number of alterations, like *ROS1* gene fusions, are emerging as treatment predictive in lung cancer broadening the cohort of patients eligible for targeted treatment [1].

Until recently, clinical analyses of treatment predictive alterations in *EGFR* and *ALK* have predominantly been performed by different single gene assays, e.g., real-time PCR or pyrosequencing, and immunohistochemistry (IHC) or fluorescence *in situ* hybridization (FISH), respectively. Given the continuous discovery of new, potentially treatment predictive alterations in lung cancer (see e.g. [1]) and a growing understanding of treatment resistance mechanisms, iterative single gene diagnostics is becoming problematic. Specifically, multiple analyses per sample increase the cost, require more input material and a longer time to generate results, in addition to the cumbersome nature of some methods (e.g. FISH). With the introduction of next generation sequencing (NGS) to the field of molecular genetics, and more recently also to the field of clinical diagnostics by allowing formalin-fixed paraffin embedded (FFPE) tissues to be screened, new possibilities exist for cost-, time- and sample efficient analysis of many different treatment predictive alterations in one analysis. Today, NGS-based diagnostics of treatment predictive mutations are running in large scale in large cancer centers worldwide and numerous reports of different implementations and techniques exist (see e.g., [2–8]). However, the technology is also increasingly introduced in smaller, often decentralized, healthcare regions at regional/local pathology departments with limitations in sample flow, budget, trained personnel, NGS equipment and bioinformatics structures, but still obliged to deliver accurate and timely results to guide patient therapy decisions.

**Figure 1:**
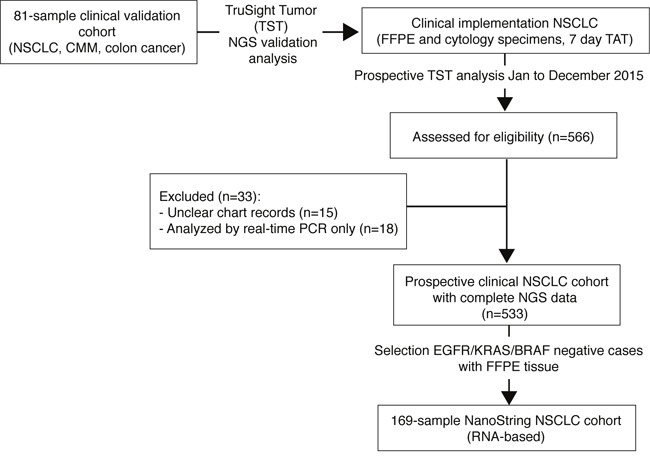
Study scheme outlining analyses and cohorts FFPE: formalin-fixed paraffin embedded tissue, TAT: turnaround time.

The aim of the present study was to: i) implement a centralized NGS-based framework in the southern health care region of Sweden, Scandinavia, corresponding to one of the larger decentralized healthcare regions in Sweden, for clinical analysis of treatment predictive mutations in NSCLC, ii) determine the potential diagnostic yield of the NGS testing based on a complete year of clinical analysis, and iii) to investigate the clinical potential of multiplexed gene fusion analysis of *ALK*, *RET*, and *ROS1* based on RNA expression (Figure 1).

## RESULTS

### Validation of an NGS-based assay compared to routine single gene diagnostics

To validate the Illumina TruSight Tumor (TST) NGS panel for clinical usage we analyzed 81 lung cancers, cutaneous malignant melanomas (CMMs) and colon cancers with existing clinical mutation data for hotspot mutations in *EGFR*, *KRAS*, *NRAS*, and *BRAF* (Table 1) (in addition to our previous validation of TST in a research setting, [9]). In total, the 81 cases harbored 29 known hotspot mutation calls and 63 calls of no mutation present for the investigated genes and loci. Of the total 92 mutation calls, concordance between previous single gene clinical testing methods and the TST assay was observed for 88 calls (96%) (Supplementary Table 1). Three of the four discordant calls were due to a variant detected by TST but not analyzed by the corresponding single gene assay. Excluding these variants implied a concordance of 99% between TST and prior clinical methods. In the remaining single discordant sample (a colon cancer), a *NRAS* c.182A>G variant (38% TST variant allele frequency, VAF) was detected by all methods, with an additional c.35G>C *KRAS* variant called by the prior clinical real-time PCR method. A reanalysis was performed using sections from the same tissue block with the prior clinical real-time PCR method, TST, pyrosequencing, and complementary real-time PCR (Qiagen Therascreen). Reanalysis with the clinical real-time PCR method again identified the *KRAS* c.35G>C variant, while pyrosequencing identified a different *KRAS* variant (c.35G>T, 5% VAF). In contrast, TST analysis and Therascreen real-time PCR analysis agreed that no variants in *KRAS* were observed. The observation of both an activating *KRAS* and *NRAS* mutation in the same tumor is unlikely, suggesting that the discrepant *KRAS* variant might represent a false positive call (supported by the low VAF from pyrosequencing, and the different variants reported by pyrosequencing and the prior clinical real-time PCR method).

**Table 1:** Clinicopathological characteristics of the validation and prospective cohorts

	Validation cohort		
	Lung cancer	CMM^A^	Colon cancer
Number of patients	40	22	19
FFPE/Cytology (%)	70/30	100/0	100/0
Number of hot spot mutation calls (mut/no mutation)	8/32	9/13	12/18
KRAS	-	-	10/8
NRAS	-	0/1	1/5
BRAF	-	9/12	1/5
EGFR	8/32	-	-

	Prospective cohort of lung cancer cases (n=533)			
	AC^B^	SqCC^B^	NSCLC-NOS^B^	Other^B^
Number of patients (%)	348 (65%)	90 (17%)	80 (15%)	15 (3%)
FFPE/Cytology (%)	79/21	84/16	47/53	60/40
Median age (years±sd)	69±9	72±8.3	70±8.5	68±7.6
Sex (female/male %)	53/47	38/62	51/49	60/40
Early stage cancer (%)	12.5%	7.8%	0%	20%
Clinical ALK analysis (n_tot_=491)	324	86	68	13
ALK FISH-positive (n)	10	0	4	0
ALK IHC-positive (n)	2	2	1	1
NanoString analyzed cases^C^ (%)	68 (50%)	54 (40%)	8 (6%)	5 (4%)

A: CMM: cutaneous malignant melanoma.

B: AC: adenocarcinoma, SqCC: squamous cell carcinoma, NSCLC-NOS: non-small cell lung cancer not otherwise specified, Other: including adenosquamous, large cell carcinoma, large cell neuroendocrine carcinoma, sarcomatoids.

C: Only cases that were *KRAS*/*EGFR*/*BRAF* mutation negative, from FFPE tissue, and with successful NanoString hybridizations are listed.

Using the Qiagen Therascreen *EGFR*, *KRAS*, and *BRAF* RGQ kits as reference methods in this study, we were able to validate detected hotspot variants down to 4% VAF from the NGS analysis in both the validation cohort and the subsequent prospective cohort in all tested cases, thus representing the effectively used limit of detection in later clinical samples (notably, a strict 10% tumor percentage cut-off was used for decision to perform clinical mutation testing at all).

### Clinical implementation of an NGS-based diagnostic framework

The clinical implementation, including personnel and budget, of the NGS-based framework is described in Supplementary Table 2. Following the clinical implementation (January 7, 2015), NGS analysis was the primary assay for routine clinical analysis of treatment predictive mutations in NSCLC and results were used to guide patient treatment. All identified mutations were reported to the diagnostic pathologist through a NGS report. During the prospective time period, only *EGFR* and *KRAS* mutation status were included in the pathological report returned to the treating clinician to guide treatment. For *ALK* fusions, the main method during the investigated time period was IHC and/or FISH. NanoString evaluation of RNA-based fusion detection was performed in parallel, but was not used to guide treatment. During the investigated time period (January 7 to December 31 2015), on average 12 suspected lung cancers were analyzed per week, of which 74.5% were FFPE sections and 25.5% cytology material. The turnaround time (TAT) for the molecular testing (DNA extraction, NGS analysis, and mutation report) was seven calendar days (i.e. five work days) in 94% of all cases analyzed during 2015, eight calendar days in 4%, and 9-10 days in 2% of cases (Supplementary Table 2). The median TAT for the entire molecular process (from clinical referral, pathological evaluations, molecular analysis, to the final clinical report) was 14 calendar days (mean=15±6 calendar days) (Supplementary Table 2). Of all originally referred suspected lung cancer cases, 4.7% could not be analyzed during the first pass through the centralized NGS laboratory due to insufficient DNA quality in the qPCR quality control step. The latter was caused by either degraded DNA, or more often by insufficient amounts of extracted DNA from the FFPE sections sent for analysis (mainly small biopsy specimens). With a few exceptions, all of these cases were however analyzed either by resampling followed by new NGS-analysis, or by a real-time PCR method (case then excluded from the prospective cohort analyzed in this study, see Figure 1). Based on this diagnostic framework, we collected 533 consecutively tested lung cancers by the TST NGS panel during 2015 to determine the diagnostic yield (Table 1). Notably, the proportions of the histological subtypes in the consecutive clinical testing cohort differ slightly from what might be expected from a Swedish population-based cohort (especially a lower proportion of squamous cell carcinomas, SqCC, 17% versus 21% based on data from the Swedish lung cancer registry). This suggests a potential selection bias between histological subtypes in the offered reflex-testing scheme. The bias could originate from the decentralized clinical management of patients in different regional hospitals within the healthcare region, coupled with a previous history of testing only adenocarcinomas.

### NGS-based clinical analysis of a consecutive lung cancer cohort

**Figure 2:**
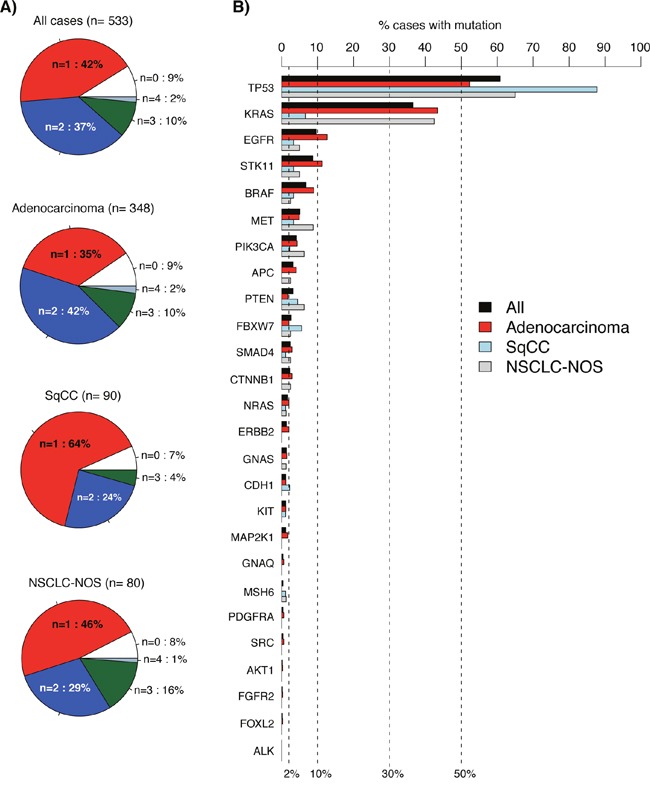
Detected variants in 533 consecutive lung cancers analyzed by the 26-gene TST panel **(A)** Pie charts of number of called variants per sample for different sample groups. **(B)** Variant frequency for the analyzed 26 genes across different sample groups (bars). Genes are ordered according to decreasing frequency in the total cohort. In A and B, all detected non-synonymous variants by the vendor supplied analysis pipeline are included.

Among the 533 cases, 889 variants were called by the standard vendor supplied data analysis pipeline. In general, analyzed cases showed few alterations in the investigated genes across different sample groups (total cohort, adenocarcinoma, SqCC, and NSCLC not otherwise specified, NSCLC-NOS), with ˜80% of cases having 1-2 called variants and 7-9% no detected variants (Figure 2A). In all major sample groups (adenocarcinoma, SqCC, and NSCLC-NOS) *TP53* was the most frequently mutated gene, while the mutational pattern for *KRAS* and *EGFR* (second and third most frequently mutated genes in total) differed between sample/histological groups (Figure 2B). For 14 genes in the 26-gene panel, mutation frequencies in the total cohort were ˜2% or less, suggesting that genetic alterations in these genes represent more rare driver events in NSCLC. Associations between mutation status for individual genes and clinicopathological variables (age, gender, and tumor histology) were scarce, with exception for *BRAF* (adenocarcinoma histology), *EGFR* (adenocarcinoma histology), *KRAS* (younger age, gender, adenocarcinoma histology), *CTNNB1* (gender), *PTEN* (adenocarcinoma histology), *STK11* (adenocarcinoma histology), and *TP53* (adenocarcinoma histology) (Supplementary Figure 1). In contrast with the literature there was no association between presence of *EGFR* mutation and gender in the total prospective clinical testing cohort (with 53/47% females/males), or in adenocarcinoma specifically (p=0.66 and 0.87, respectively, Fisher's exact test). In lack of complete patient smoking status (not consistently available in pathological referrals) this insignificant association is difficult to assess.

*KRAS* and *EGFR* (first and second most mutated oncogenes) showed a striking enrichment of specific, well-established, activating variants. In *KRAS*, variants in codons 12 and 13 constituted ˜91% of all detected variants, while exon 19 deletions and p.L858R point mutations constituted ˜75% of all detected variants in *EGFR* (see Supplementary Figure 2 for complete protein localization of variants in *EGFR*, *KRAS*, *BRAF* and *TP53*). For *EGFR*, mutations beside exon 19 deletions and p.L858R (c.2573T>G) variants, such as p.G719X (c.2155G>A, c.2156G>C, and c.2156G>T), p.L861Q (c.2582T>A, n=3), and exon 20 insertions (n=2), each represented ≤5% of the total *EGFR* mutation spectrum. *EGFR* p.T790M resistance mutations (n=4) were only observed in cases re-biopsied after progression on targeted *EGFR* treatment. In these cases, the originally detected activating *EGFR* mutation (e.g. p.L858R) was always present, while the p.T790M alterations always showed lower VAFs in each case suggesting tumor heterogeneity (5.4-12.2% VAF versus 8.1-88% VAF for activating mutations).

For *BRAF* (the third most mutated oncogene), a slightly higher variability in detected variants was observed compared to *EGFR* and *KRAS*. This included both codon 600 (38% of *BRAF* variants, 3.7% of adenocarcinomas, and 1.1% of SqCCs specifically) and codon 601 (5.4% of *BRAF* variants) variants known or suggested to be treatment predictive in malignant melanoma, but also variants in codons 466 (11%), 469 (5.4%), and 594 (22% of *BRAF* variants) for which the treatment predictive value to BRAF inhibitors are not fully elucidated (Supplementary Figure 2). *BRAF* variants were more often found in older patients (>60 years), with only 13.5% of all detected variants in patients younger than 60 years.

### NanoString ALK, RET, and ROS1 gene fusion analysis

The ability of the NanoString assay to identify gene fusions in *ALK*, *RET* and *ROS1* was first successfully validated in four cell lines with known fusion gene rearrangements, HCC78 (*ROS1*-*SLC34A2*), KARPAS-299 (*ALK*-*NPM1*), LC-2/ad (*CCDC6*-*RET*), and H2228 (*EML4*-*ALK*). Next, gene fusion analysis was performed on RNA from FFPE tissue from 169 lung cancers in the prospective 533-sample cohort, which did not harbor mutations in *EGFR*, *KRAS* or *BRAF* (referred to as triple-negative cases hereon) (Figure 1). NanoString analysis was restricted to cases with FFPE tissue, as the clinical handling of cytology specimens at local pathology departments did not include combined RNA and DNA extraction. Of the 169 analyzed cases (representing 87% of all triple-negative FFPE cases in the 533-sample cohort), 34 hybridizations (20%) were deemed as failures based on too low signals from included housekeeping genes. The failure of these FFPE cases is likely due to extensive RNA degradation in the tissue blocks caused by the fixation process. During 2015, there was no standardization of the time for formalin fixation between different pathology departments in the healthcare region.

Interestingly, the proportion of inclusive NanoString cases was equivalent to the 17% of cases with an inconclusive ALK status by IHC and/or FISH in the total clinical cohort. However, there was no significant association between an inconclusive ALK IHC/FISH call with an inconclusive NanoString call (p=0.78, Fisher's exact test), suggesting that: i) different degradation processes and/or technical issues are in action, and ii) that the methods may complement each other in detecting gene fusion events.

**Figure 3:**
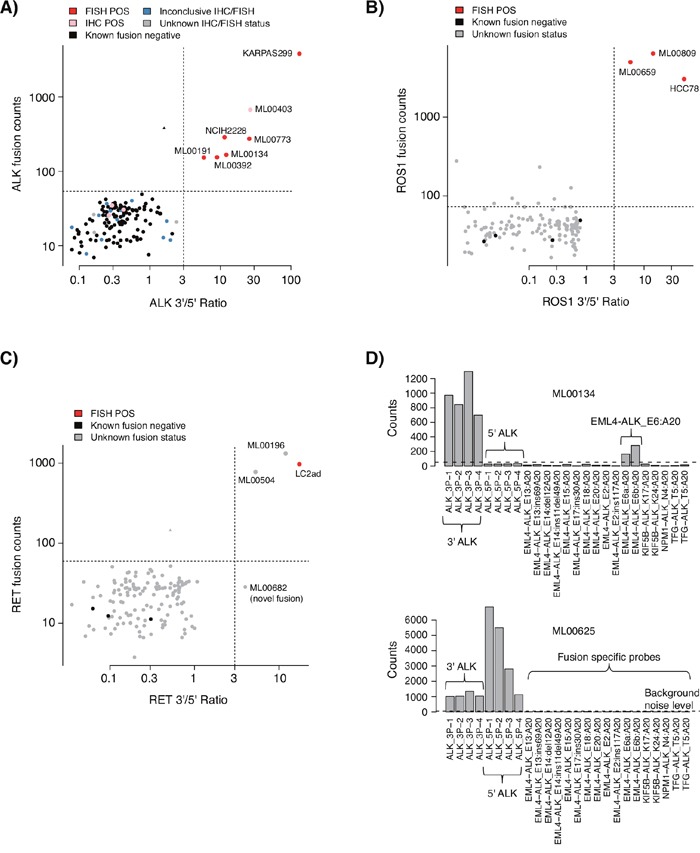
NanoString gene fusion analysis of 135 *EGFR/KRAS/BRAF* mutation negative tumors from a consecutive 533 NSCLC cohort **(A)** NanoString *ALK* gene fusion analysis of 135 tumors from the consecutive prospective cohort, and four cancer cell line controls included for reference. **(B)** NanoString *ROS1* gene fusion analysis of the 135 tumors and four cell lines. **(C)** NanoString *RET* gene fusion analysis of the 135 tumors and four cell lines. One sample, ML00682 (lower right quadrant), displays a high 3’/5’ ratio but no elevated fusion specific signal, suggesting a fusion not included in the fusion specific probe set. In A to C, analyses were performed as described in Lira et al. [15], using the same thresholds (dotted horizontal and vertical lines). Briefly, based on the dual plotting of a gene fusion specific signal and the 3’/5’ expression ratio of probes located around the tyrosine kinase exon, a gene fusion positive case with a known fusion should be located in the upper right quadrant, while a gene fusion positive case without an included fusion specific probe should be located in the lower right quadrant. Negative cases should be located in the lower left quadrant. **(D)** Top panel shows an example of an *ALK* gene fusion positive adenocarcinoma identified by both FISH and NanoString analysis. Bottom panel shows a LCNEC case with a positive ALK IHC call, but inconclusive FISH, that do not display any fusion event based on NanoString analysis. The latter is based on the simultaneously high 3’ and 5’ expression of probes around the tyrosine kinase exon in the *ALK* gene (case corresponds to a pink labeled sample in panel A).

Among the 135 triple-negative cases successfully analyzed by the NanoString assay, gene fusions were detected in ten cases (7.4%); five (3.7%) *ALK* gene fusions (four EML4-ALK_E13:A20 and one EML4-ALK_E6ab:A20 fusion), three (2.2%) *RET* fusions (two *CCDC6-RET_C1:R12* and one novel fusion), and two (1.5%) *ROS1* fusions (*SLC34A2-ROS1_S4:R32* and *SDC4-ROS1_S2:R32*) (Figures 3A-3C). All cases harboring gene fusions were adenocarcinomas, corresponding to 15% of the 67 analyzed triple-negative adenocarcinomas, consistent with the literature [10]. All NanoString called *ALK* and *ROS1* fusions were confirmed by clinical IHC and/or FISH data, and no ALK fusion positive case identified by FISH was missed by the NanoString assay. Three non-adenocarcinoma cases (two SqCC and one large cell neuroendocrine tumor) with positive ALK IHC staining, but inconclusive FISH calls, did not show gene fusions in the NanoString analysis (Figure 3A, pink labeled cases, lower left quadrant, all with tumor cell content >70%). Notably, all three cases showed high expression of both 3’ and 5’ probes of the ALK tyrosine kinase domain in the NanoString data (indicating lack of gene rearrangement, Figure 3D), suggesting that these ALK IHC stainings could represent false positive gene fusion calls (although treatment data is required to fully confirm such a hypothesis). Interestingly, despite their ALK IHC positive staining none of these three patients have so far received anti ALK therapy in the clinic.

In one sample we detected a probable *RET* fusion through the 3’/5’ NanoString ratio not targeted by a fusion specific NanoString probe (Figure 3C, ML00682). Complementary experimental RNA-based NGS analysis (ArcherDX, Boulder, CO, US), performed as previously described [9], identified the suspected fusion to be a *TRIM24-RET* fusion, confirming the NanoString assay's ability to detect also novel fusions.

### Co-occurrence of actionable mutations and gene fusions in a consecutive cohort of lung cancers referred to mutation and gene fusion screening

Integration of TST mutation data, ALK IHC/FISH, and NanoString *ALK*, *RET*, and *ROS1* gene fusion analysis for the complete 533-sample prospective cohort is shown in Supplementary Figure 3.

**Figure 4:**
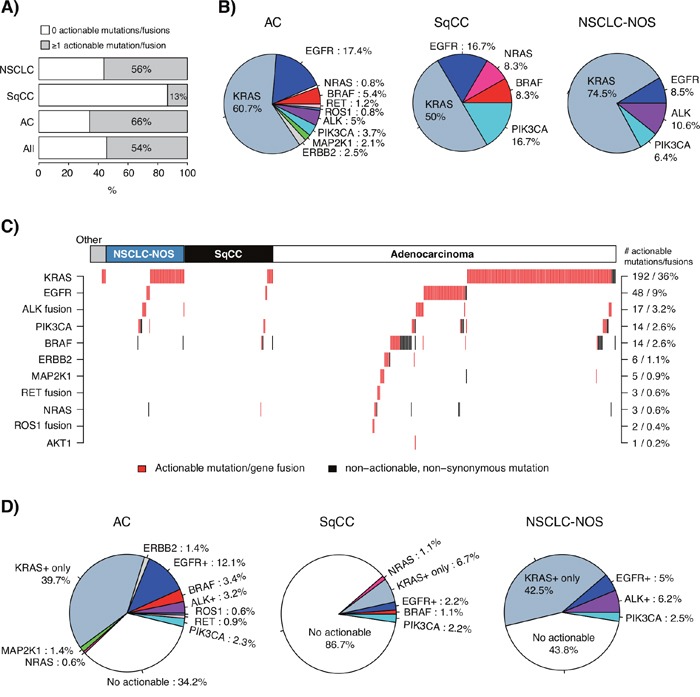
Integration of actionable mutations and gene fusions in the consecutive 533-sample cohort **(A)** Proportion of cases with ≥1 actionable alteration in the total 533-sample cohort, adenocarcinomas (AC), SqCCs, and NSCLC-NOS. **(B)** Distribution of detected actionable variants according to the gene in which they fall for adenocarcinomas (AC, n=242 detected variants), SqCCs (n=12 variants) and NSCLC-NOS (n=47 variants). **(C)** Heatmap describing defined actionable and non-actionable non-synonymous variants and gene fusions in investigated genes identified in each case. Each column represents a sample; each row represents a gene. Numbers and proportions displayed on the right axis correspond to the total cohort (533 samples). **(D)** Proportion of cases with actionable mutations in adenocarcinoma (AC, n=348 samples), SqCC (n=90 samples), and NSCLC-NOS (n=80 samples). In each pie chart, EGFR+ corresponds to the proportion of cases with an actionable *EGFR* mutation irrespective of other alterations, ALK+ corresponds to cases with an *ALK* gene fusion irrespective of other alterations, and KRAS+_only corresponds to cases with only an actionable *KRAS* mutation. Consequently, some *BRAF* mutated cases may for instance harbor also an actionable *KRAS* variant. In all panels, not all cases were analyzed for gene fusions by the NanoString assay; consequently these estimates (mainly *ROS1* and *RET*) should be interpreted as low frequency proportions.

To investigate the potential additional clinical yield of a combined TST and multiplexed gene fusion assay (NanoString) compared to the current targeted therapy options in the health care region (EGFR and ALK inhibitor treatment), we analyzed actionable alterations defined from the literature in the 533-sample cohort. First, we defined a set of both acknowledged and proposed actionable oncogene mutations in specific oncogenes (*KRAS*, *EGFR*, *BRAF*, *PIK3CA*, *NRAS*, *ERBB2*, *MAP2K1*, and *AKT1*) in addition to *ALK*, *RET*, and *ROS1* gene fusions, using information from public sources [11] and reported studies [2, 12]. Next, we stratified the 533-sample cohort based on existence of these actionable alterations in individual cases, finding that 54% of all cases, 66% of adenocarcinomas, 13% of SqCCs and 56% of NSCLC-NOS harbored ≥1 alteration (Figure 4A). Of these actionable variants, alterations in *KRAS* dominated in all subgroups, followed by *EGFR*, *BRAF* and *PIK3CA* (Figure 4B). Gene fusions accounted for 7% of actionable alterations in adenocarcinomas. While the majority of detected actionable variants appeared mutually exclusive across samples (Figure 4C), a number of cases showed multiple actionable variants, e.g., concurrent *KRAS* and *PIK3CA* mutations, concurrent *KRAS* and *BRAF* mutations, and concurrent *KRAS*/*EGFR*/*BRAF* mutations and *ALK* fusions. While the two former observations may be explained by tumor subclonality, the high proportion of the latter observation is intriguing given the reported near to mutual exclusiveness of these alterations [10, 13]. Possible explanations may be tumor subclonality, however technical/interpretation issues in the ALK diagnostic scheme cannot be excluded.

Second, we sought to determine the subset of patients with different histological subtypes that could be eligible for potential emerging treatments based on the defined actionable alterations (Figure 4D). In adenocarcinoma, this analysis suggested that 10.6% (50.3% if including *KRAS*) of cases could be eligible for emerging targeted treatments, beyond the 15.3% of cases eligible for standard EGFR or ALK targeted therapy (Figure 4D). For SqCC, similar proportions were lower, 4.4% of cases (11.1% if including *KRAS*) could be eligible for emerging treatments, in addition to the 2.2% eligible for EGFR targeted therapy. Finally, for NSCLC-NOS 2.5% of cases (45% with *KRAS*) could be eligible for novel/emerging targeted treatments, in addition to the 11.2% of cases eligible for standard EGFR or ALK targeted therapy.

## DISCUSSION

In the era of personalized medicine accurate multi-gene diagnostics is crucial. In the present study, we describe the clinical implementation of an NGS-based diagnostic framework and a parallel validation of a RNA based gene fusion assay for analysis of treatment predictive alterations in a prospective and consecutive clinical testing cohort of mainly advanced NSCLC patients analyzed during a single year within a regional health care region in a Nordic country (Sweden).

Together with several recent reports [2–8, 14–16], our clinical validation and implementation of a commercial DNA amplicon-based NGS assay support the usage of this technique in routine clinical diagnostics of NSCLC compared to previous single gene diagnostics also in smaller regional healthcare regions, based on concordance between techniques (99% in this study), turnaround time, sample success rate over time, accuracy, limit of detection, and cost. In agreement with both Fisher et al. [5] and Hagemann et al. [2], the success rate and clinical feasibility of our NGS framework is highly dependent on central pathological review by experienced diagnostic pathologists together with standardized and quality controlled tissue handling, to ensure sufficiently high proportions of malignant cells in specimens with adequate nucleic acid quality. A challenge for regional/county hospitals may be the bioinformatics aspect of NGS. Using the TST vendor-supplied bioinformatics pipeline we were able to detect and validate by orthogonal methods known activating driver mutations in *EGFR*, *KRAS* and *BRAF* below 5% VAF in cases with ≥10% tumor cell content by routine pathological assessment. This sensitivity was especially important for analysis of *EGFR* p.T790M mutations in patients undergone re-biopsy after progression on first generation EGFR inhibitors. For all such patients, we observed tumor subclonality (inferred based on differences in VAF) between p.T790M mutations and original activating mutations (p.T790M always with a lower VAF). In agreement with Fisher et al. [5], we did observe cases where the vendor-supplied bioinformatics pipeline failed to adequately annotate complicated insertions and deletions in, e.g., *EGFR* (exon 19 deletions and exon 20 insertions), calling for continuous development of these pipelines and/or usage or orthogonal data analysis protocols. Detection of larger insertions and deletions is challenging using amplicon-based techniques, especially in cases with low tumor cell content or tumor subclonality. In our consecutive 533-sample cohort we identified *EGFR* exon 19 deletions down to 8% VAF, and participation in the ESP Lung Quality Assessment Scheme [17] and analysis of samples referred to testing after December 2015 also confirmed detection of *EGFR* exon 20 insertions down to 7% VAF. We believe these findings show that while additional work is needed for challenging indels, vendor supplied analysis pipelines to us appear adequately robust and sensitive for routine clinical use in regional healthcare units lacking strong bioinformatical infrastructure.

The low number of detected variants per sample in this study is consistent with similar targeted NGS-based reports [5, 16] and the gene driver selection process and pan-cancer approach of TST and similar gene panels (like the Ion Torrent AmpliSeq Colon and Lung panel). Ethnicity plays a role in the prevalence of certain genetic markers in NSCLC [18]. For several of the investigated genes (e.g. *TP53*, *PTEN*, *EGFR*, *KRAS*, *ERBB2*) the observed mutation patterns and frequencies in our Swedish cohort agree with previous reports on clinical patient cohorts (predominantly comprising of advanced cancers) of similar ethnicity and/or geographic origin (Scandinavia) [19–25], but also with cohorts consisting of selected non-consecutive patients with operable disease [26, 27]. Alteration frequencies in the NSCLC-NOS subgroup are difficult to interpret and compare, as this subgroup comprises of a mixture of different histological subtypes (a majority is expected to be adenocarcinoma) due to mainly insufficient tissue material (>50% of NSCLC-NOS cases were cytology specimens) that precluded comprehensive histological subtyping by IHC. A few notable discrepancies in our cohort are apparent. For *PIK3CA*, we observe a considerably lower mutation rate (only 2%) in SqCC cases compared with literature reports of 7-16% [16, 19, 26]. The cause of this difference is difficult to determine without extensive comparison of the tested clinical SqCC cohort versus a more population-based cohort from our region. For *BRAF*, we observe a high general mutation frequency in adenocarcinomas (9%), with a 3.7% V600 mutation rate. While the overall mutation frequency is clearly higher compared to some recent studies [19, 28, 29], it is in line with others using e.g. the Ion Torrent AmpliSeq Colon and Lung panel [16]. Consistently, the proportion of specific V600 alterations was slightly higher in our clinical testing cohort than previous literature reports [19, 28, 29] (3.7% versus ˜2%).

The main purpose of NGS (and multiplexed gene fusion assays in general) in the clinic is to increase the list of actionable variants for a patient, without increasing the cost, time and tissue requirement compared to serial single gene testing. In addition, occurrence and/or co-occurrence of mutations in tumor suppressor genes like *TP53* and *STK11* with typical oncogene driver mutations in lung cancer have been suggested to have implications for prognosis and treatment response [30–32], which may be of complimentary clinical value. Our analysis of 533 consecutive NSCLCs screened during a single year showed considerable differences between histological subgroups in the proportion of cases harboring a known or suggested actionable variant, with adenocarcinomas having the greatest potential benefit from this type of analyses (Figure 4). We acknowledge that inclusion of *KRAS* as an actionable gene in this type of analysis is not unproblematic (and hence we report frequencies with and without *KRAS*). For SqCC, results must be interpreted with great care given the small number of cases and individual mutations. Irrespectively, despite the individually low frequency of many potentially actionable variants defined in the current study (e.g. *ERBB2*, *BRAF*, *RET*, *ROS1*, *PIK3CA*), the high incidence of lung cancer implies that a large population worldwide is affected, supporting clinical trials or routine molecular screening programs in the disease [19].

*ROS1* gene fusions have been shown to be treatment predictive for ALK-inhibitor drugs [33]. In our investigated cohort of triple-negative NSCLCs (*EGFR*, *KRAS*, and *BRAF* mutation negative) the number of cases with either *ROS1* or *RET* fusions were similar to that of ALK-positive cases, supporting the need of multiplexed gene fusion diagnostics in NSCLC and adenocarcinoma specifically. For the non-adenocarcinoma cases that were ALK positive by IHC in the triple-negative cohort, NanoString analysis suggested overexpression of the entire gene by some other mechanism than gene rearrangement (see, e.g., Figure 3D). This more detailed view of gene fusion events supports the usage of multiplexed methods like NanoString as a complementary orthogonal method, or even replacement, for IHC/FISH when possible. Moreover, due to the flexibility and capacity of the NanoString technology, additional gene fusions as well as *MET* exon 14 skipping events can easily be added in a design update (see e.g. [34]). Finally, the experimental TAT for the NanoString assay may be very short, potentially down to three working days including nucleic acid extraction (Supplementary Table 2).

While the introduction of NGS in the clinical setting represents a major leap forward; current commercial amplicon-based panels (e.g. the TST and Ion Torrent Ampliseq Colon and Lung panels) are biased towards analyzing hotspot alterations in a limited set of oncogenes often selected through a pan-cancer approach. To some extent, these panels offer the possibility to detect intrinsic or acquired resistance mechanisms to targeted treatment, mainly p.T790M (as shown in this study) and p.C797S mutations in *EGFR* (first to third generation inhibitors) and specific gatekeeper mutations in *ALK* (like p.L1196M and p.G1269A), in patients re-biopsied after treatment failure. However, most panels (including TST and frequently used Ion Torrent panels) are less well suited to detect EGFR/ALK resistance mechanisms caused by alterations in other genes. Here, panel design and size constraints, but also problems in calling copy number alterations (like *MET* amplification as a mechanism of resistance to EGFR inhibitors) reliably in tumors with considerable non-malignant infiltration represent limiting factors. Therefore, diagnostic platforms based on, e.g., hybrid capture methods of either DNA alone (see e.g. [35, 36]) or DNA and RNA combinations (e.g. the Illumina TruSight Tumor 170 panel and the AmpliSeq based Thermo Fisher Oncomine™ Focus/Comprehensive panels) that allow considerably more sequence to be analyzed could be the next preferable step also outside large comprehensive cancer centers. These assays could allow simultaneous detection of mutations, gene fusions, and copy number alterations (like drug targetable *MET* and *FGFR1* amplifications) in a large number of genes. However, considering the observed failure rate of 20% for the NanoString RNA gene fusion assay in our prospective clinical samples, it remains to be shown that RNAseq approaches can do better in daily clinical practice. Finally, while tissue-based diagnostics is the cornerstone of diagnostic tumor pathology today, less invasive sampling methods like blood-based assays targeting e.g. circulating tumor DNA, or analysis of exhaled breath condensates [37, 38] are increasingly gaining interest as they could facilitate a more active and less invasive treatment monitoring. However, these applications may require more sensitive sequencing techniques, different logistics, and optimized sample preparations than presently used in most local diagnostic pathology departments.

## MATERIALS AND METHODS

### Ethics statement

The study was approved by the Regional Ethical Review Board in Lund, Sweden (Registration no. 2014/748 and 2015/575). By decision of the Ethical Review Board, specific written informed consent from patients were not required. No personal data was used for this study. In accordance with the decision of the Ethical Review Board, patients were informed about the study through local advertisement in news media in the region.

### Tumor validation cohort

A tumor cohort comprising of 40 NSCLCs, 22 CMMs, and 19 colon cancers with available *BRAF*, *KRAS*, *NRAS*, and/or *EGFR* mutational data from routine clinical analysis within the southern health care region of Sweden using single gene assays (see below) were collected (Table 1). Tissue types from included patient tumors included routine FFPE sections from resected material or needle biopsies (typically 6×5um sections), sections from cytology cellblocks (typically 10×5um or 10×10um sections), or DNA extracted from cytology slides (see below).

### Consecutive prospective tumor cohort

In the southern Swedish health care region (comprising close to 1.8 million inhabitants), ˜800 new lung cancer cases (of any stage and histology) are identified annually. A consecutive prospective clinical testing cohort of 533 lung cancers, representing 526 unique patients, subjected to routine NGS-based mutational analysis within the southern health care region of Sweden, including two university-affiliated and four regional pathology departments, with additional samples from a third university-affiliated pathology department outside the healthcare region were collected between January 7 to December 31 2015 (Table 1, Figure 1). During this period a reflex testing procedure was allowed in the health care region, meaning that all lung cancers that were not SCLC or carcinoids could be sent for clinical mutation testing, including also some early stage tumors. All cases were analyzed at an established central NGS laboratory within the Division of Oncology and Pathology, Department of Clinical Sciences Lund, Lund University through the Center for Molecular Diagnostics (www.skane.se/cmd). All identified mutations were reported back to the diagnostic pathologists in a molecular report by the central NGS laboratory. During the time period, only actionable mutations in *EGFR* and hotspot mutations in *KRAS* were included in the final clinical report (signed by a diagnostic pathologist) returned to the treating clinician. Data on lung cancer histology and tumor stage was obtained from patient charts and in accordance with the classification scheme used at the time of diagnosis. Tissue sources included primary lung tumors, lymph node metastases, or extranodal distant metastases. Sample types from included patient tumors were either tissue blocks or cytology specimens.

### Tissue selection for routine clinical mutation analysis

Tumor morphology was determined by the clinical pathologist. In cases with apparent keratinizing SqCC, IHC was normally not performed. In case of morphologically apparent adenocarcinoma, the standard immunohistochemical panel included at least TTF-1, while in case of NSCLC without clear morphology the panel included at least TTF-1 and either CK5 or p40. If these markers were negative, further stains including CK7 or a broad cytokeratin were performed. Also, the morphological appearance, patient history and clinical and radiological findings guided the initial selection of stainings. Neuroendocrine markers were added in cases with neuroendocrine morphology. If a diagnosis of primary lung cancer was uncertain, or if the DNA content and/or quality was to low for NGS-analysis (requiring real-time PCR analysis), the case was excluded from this study (Figure 1). During the study period, encountered cases of pulmonary adenocarcinoma, SqCC, adenosquamous carcinoma, sarcomatoid carcinoma, NSCLC-NOS, large cell carcinoma and LCNEC based on biopsy or cytology were unselectively tested for predictive molecular alterations.

The suitability of a material for mutational analysis was assessed by a pathologist based on hematoxylin and eosin (H&E) stains of archived FFPE tissue blocks and/or cytology specimens. A representative area with high frequency of malignant cells was identified, from which sections for mutational analysis was taken followed by new H&E sections to ensure that a representative material had been taken. An estimate of tumor cell content was made by a diagnostic pathologist, with a requirement of ≥10% for the mutational analysis. In addition to FFPE tissue blocks, tissue material for mutation analysis could also originate from cytology slides, or sections from centrifuged and paraffin embedded cytology material (cell blocks). Sections were stored at -20°C until nucleic acid extraction, due to logistical batching with frozen DNA aliquots from cytology specimens.

In case of preparation of cell lysate from cytology slides, a representative tumor cell rich area of a cytology slide was identified, the slide was scanned (to enable future clinical review), and the glass cover slip was removed using xylene followed by a rehydration step in ethanol. Thereafter, the cells were lysed using 180ul ATL Buffer from Qiagen (Qiagen, Hilden, Germany). DNA was extracted from the lysate within 24h and stored at -20°C.

### DNA and RNA extraction

DNA and RNA for NGS-based mutation analysis and NanoString (Seattle, WA, US) gene fusion analysis were extracted using the Qiagen AllPrep kit for FFPE tissue and automated on the QIAcube instrument (Qiagen). The protocol was modified with an extended proteinase K digestion (overnight) for the DNA extraction to obtain higher DNA yields. DNA from cytology slides was extracted using the QiaAmp DNA Micro kit (Qiagen). RNA was not extracted from cytology specimens, as these extractions were not performed at the central NGS laboratory in contrast to FFPE extractions.

### Mutational validation techniques

Mutational status for hotspot mutations in *NRAS*, *KRAS*, *BRAF*, and *EGFR* were obtained for the validation cohort using pyrosequencing for *EGFR* or real-time PCR for *KRAS* and *NRAS* (Entrogen, Woodland Hills, CA, US) and *BRAF* (Qiagen RGQ Therascreen®) performed and validated for routine clinical diagnostics within the health care region (Region Skåne, Sweden). Independent validation analysis of NGS results for: i) samples with very low variant allele frequencies (VAFs) in *EGFR*, *KRAS*, or *BRAF* (VAF <5%) or ii) randomly selected *EGFR* and *KRAS* mutation negative cases on a regular basis was performed using Qiagen Therascreen® RGQ PCR Kits for *EGFR*, *KRAS*, and *BRAF* according to the manufacturer's protocol.

### NGS-based mutational analysis

NGS-based mutation analysis was performed using the Illumina TST panel on a MiSeq instrument according to manufacturer's instructions (Illumina, San Diego, CA, US). The TST panel is an exon-focused panel, allowing theoretical identification of all variants in screened exons, opposed to a specific hotspot mutation panel. Analyzed regions included a selected set of complete exons in 26 genes: *AKT1* (exon 2), *ALK* (exon 23), *APC* (exon 15), *BRAF* (exons 11, 15), *CDH1* (exons 8, 9, 12), *CTNNB1* (exon 2), *EGFR* (exons 18, 19, 20, 21), *ERBB2* (exon 20), *FBXW7* (exons 7, 8, 9, 10, 11), *FGFR2* (exon 6), *FOXL2* (exon 1), *GNAQ* (exons 4, 5, 6), *GNAS* (exons 6, 8), *KIT* (exons 9, 11, 13, 17, 18), *KRAS* (exons 1, 2, 3, 4), *MAP2K1* (exon 2), *MET* (exons 1, 4, 13, 15, 16, 17, 18, 20), *MSH6* (exons 5), *NRAS* (exons 1, 2, 3, 4), *PDGFRA* (exons 11, 13, 17), *PIK3CA* (exons 1, 2, 7, 9, 20), *PTEN* (exons 1, 2, 3, 4, 5, 6, 7, 9), *SMAD4* (exons 8, 11), *SRC* (exon 10), *STK11* (exons 1, 4, 6, 8), and *TP53* (exons 2, 3, 4, 5, 6, 7, 8, 9, 10, 11). Prior to library preparation a quality control qPCR assay was performed as described in the TST instructions. In the TST assay, sample DNA amount is not fixed. Instead, the quality control assay determines a sample volume used as assay input (maximum of 2×10ul) based on a calculated delta Ct value (higher values implies poorer DNA). Thus, actual DNA input in the NGS assay may vary dramatically between samples of high quality (e.g. 16x dilution) to samples with low quality (no dilution). Routinely, samples with a quality score of 7-8 could be analyzed by NGS (recommended Illumina upper threshold was 6). Samples with higher delta Ct scores were directly assayed by the Qiagen EGFR Therascreen assay to reduce the number of inconclusive NGS runs. Four to six samples were multiplexed using the Illumina V2 sequencing chemistry, while 7-12 samples were multiplexed if using the V3 sequencing chemistry. Alignment, quality filtering, variant calling, and variant annotation were performed as described [9], using the vendor supplied data analysis pipeline. Base coverage >1000X were used as a sequencing quality control threshold for variant calling. Limit of detection for variants were not fixed in percentage, as the main variant filtering step in the variant calling was the requirement of occurrence of a variant in both TST library pools for a sample (seewww.illumina.com for explanation of the bidirectional design of the TST assay). Effectively, a limit of detection of 4% were set in the clinical context, as all such hotspot variants in *EGFR*, *BRAF*, and *KRAS* could be validated by a real-time PCR assay. Detected *TP53* variants were screened against the IARC database [39], with no variants being annotated as a known polymorphism, and 95% of annotated variants being considered as deleterious by both the AVGVD and SIFT prediction tools.

Actionable mutations (defined in [2, 11, 12]) in *KRAS* (codon 12, 13, and 61 variants), *EGFR* (exon 19 deletion, exon 20 insertion, T790M, codon 719 (exon 18), 858, and 861 variants), *BRAF* (codon 600 variants), *PIK3CA* (codon 542, 545, 1047, and 1047 variants), *NRAS* (codon 12, 13, and 61), *ERBB2* (exon 20 insertions), *MAP2K1* (codon 56 and 57 variants), and *AKT1* (L52R variant) and gene fusions in *ALK*, *RET*, and *ROS1* were summarized for each sample.

### NanoString gene fusion analysis

Analysis of *ALK*, *RET*, and *ROS1* gene fusions in FFPE tissue were performed using a RNA-based NanoString nCounter Elements assay. For each gene, a probe set was designed using the approach reported in Lira et al. [15] using two sequence-specific probe cocktails consisting of a mixture of 5’ capture and 3’ reporter probes with a target specific sequence. In addition to the 3’ 5’ approach, fusion specific target probes spanning the known exon-exon junction of fusion transcripts in the *ALK*, *RET* and *ROS1* genes were added based on the toehold approach established by NanoString and reported by Zhang et al. [40] (see this study for exact listing of specific fusions analyzed). This dual design allows gene fusions to be detected by the 3’/5’ ratio difference alone (if the specific gene fusions is not included among the toehold designed probes), or by both the 3’ 5’ probes and expression of a specific toehold probe (see Lira et al. [15] for details). All probes where synthesized by Integrated DNA Technology (IDT Inc., Coralville, USA). A RNA pool of the HCC78 (*ROS1*-*SLC34A2*), KARPAS-299 (*ALK*-*NPM1*), LC-2/ad (*CCDC6*-*RET*), and H2228 (*EML4*-*ALK*) cell lines were used as controls on all NanoString Elements cartridges, and prepared as described [9]. 100-250 ng of total RNA was hybridized for each sample for 24h at 67°C. Data analysis, including background correction, scaling based on positive controls, calculation of 3’/5’ fusion ratios, and thresholds for calling gene fusions were performed/used as described by Lira et al. [15] using the R statistical language [41]. An analysis was called as failure if its H/H_i_ ratio as described by Lira et al. [42] was >8. In a series of dilution experiments using clinical tumors with different gene fusions and pathologically estimated tumor percentages, we estimated the limit of detection to be at least 5% for the NanoString assay, i.e., the assay may detect a fusion in a sample with ≥5% tumor cells mainly due to the order of expression of a gene fusion on the RNA level.

### ALK and ROS1 IHC and/or FISH analyses

ALK IHC and/or *ALK* FISH data was available for 98.2% of all NanoString analyzed cases as part of the routine clinical diagnostic scheme in lung cancer within the health care region. ALK IHC was performed using the D5F3 antibody (Ventana Medical Systems, Tucson, AZ, US), and *ALK* FISH analysis using the Vysis ALK break apart FISH probe (Vysis, Abbot molecular, Des Plaines, IL, US) according to manufacturers’ instructions. *ROS1* gene fusions were validated using the Vysis ROS1 break apart FISH probe according to manufacturers’ instructions. *RET* fusions were not validated by FISH, as no validated assay was available in collaborating pathology departments.

## CONCLUSIONS

The present study describes a clinical implementation of NGS-based diagnostics for analysis of treatment predictive mutations in NSCLC, demonstrating that such methods can be incorporated into daily clinical practice in regional healthcare regions with constraints in budget, personnel and infrastructure. Although mutation profiles in our prospective Swedish cohort, comprising mainly of advanced stage patients, does not differ considerably from other Western patients some differences exist. Importantly, multiplexed gene diagnostics provide information for both current and emerging treatments, as well as insights into mechanisms of treatment resistance to targeted therapy. In order to allow a more personalized cancer care for lung cancer patients, innovative clinical trials and programs should take advantage of improvements in clinical diagnostics through these multigene assays to determine their actual clinical benefit.

## Abbreviations

CMM: cutaneous malignant melanoma
FFPE: formalin-fixed paraffin embedded
FISH: fluorescence *in situ* hybridization
LCNEC: large cell neuroendocrine carcinoma
NGS: next generation sequencing
NSCLC: non-small cell lung cancer
NSCLC-NOS: non-small cell lung cancer not otherwise specified
SCLC: small cell lung cancer
SqCC: squamous cell carcinoma
TAT: turnaround time
TST: TruSight Tumor
VAF: variant allele frequency
