# Supplementary material for: Clinical framework for next generation sequencing based analysis of treatment predictive mutations and multiplexed gene fusion detection in non-small cell lung cancer
Source: Oncotarget. 2017 Mar 16;8(21):34796–810. doi: 10.18632/oncotarget.16276 (PMC5471012; doi:10.18632/oncotarget.16276)
Supplement: Supplementary file 1 [file oncotarget-08-34796-s001.pdf]

Clinical framework for next generation sequencing based analysis of treatment predictive mutations and multiplexed gene fusion detection in non-small cell lung cancer

SUPPLEMENTARY MATERIALS

SUPPLEMENTARY DATA

Supplementary Table 1. Word table describing the concordance between NGS mutation data and existing clinical diagnostics for the validation cohort.

Supplementary Figure 1. PDF figure (with legend) describing the clinical implementation of the NGS diagnostic framework.

Supplementary Table 2. Excel table, describing associations between NGS mutation data and clinicopathological variables for the consecutive 533-sample cohort.

Supplementary Figure 2. PDF figure (with legend) describing the localization of detected mutations in *KRAS*, *EGFR*, *BRAF* and *TP53* in 533 consecutive NSCLC cases.

Supplementary Figure 3. PDF figure (with legend) describing the complete pattern of detected gene fusions and non-synonymous mutations in the 533-sample prospective cohort.

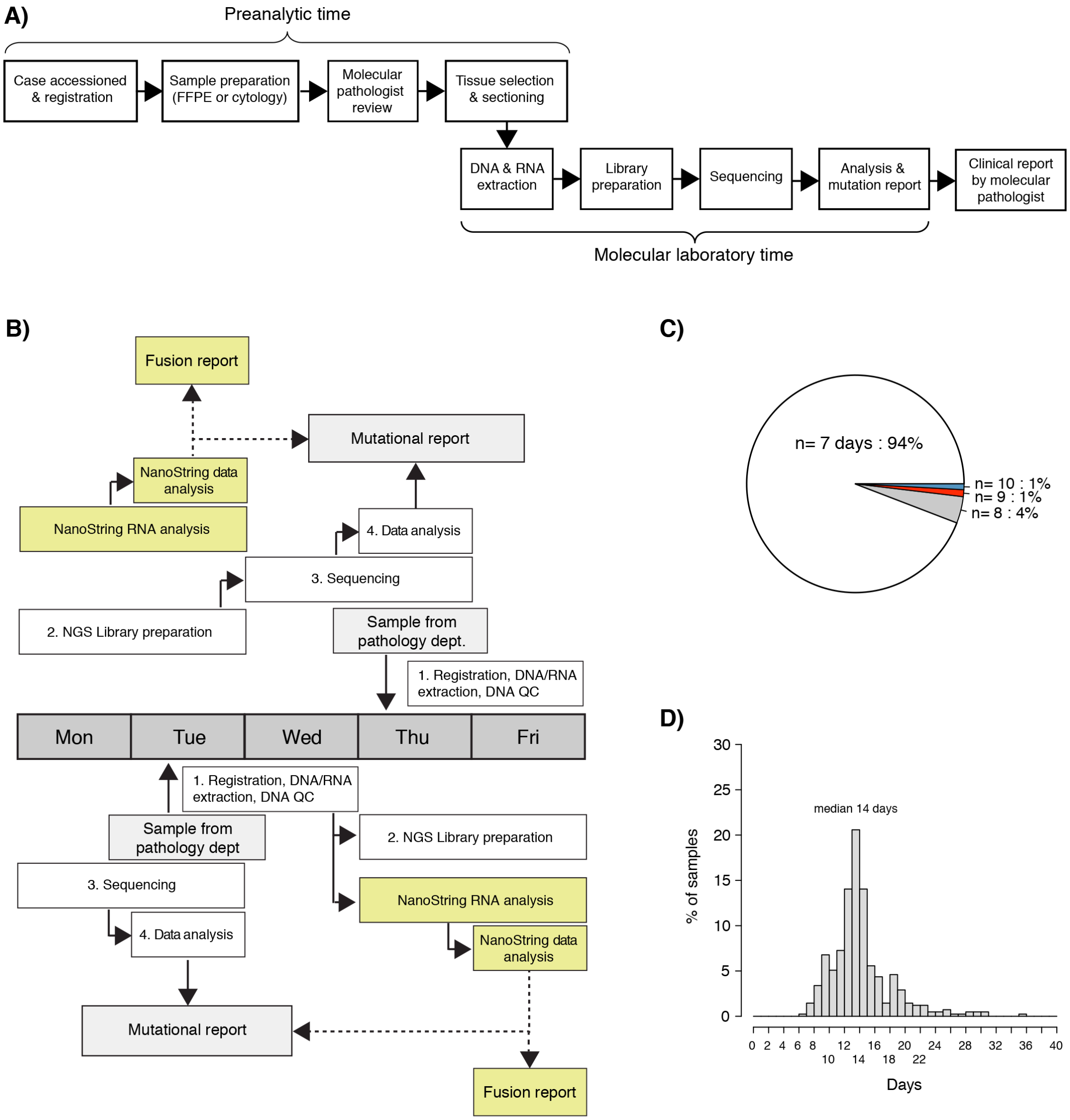

Human resources (corresponding to full time positions):
- 3 laboratory technicians
- 1 Molecular biologist / scientist (lab manager)

Total clinical cost (Reagents, staff, laboratory, analysis): 730$ / sample (2016)

Larger laboratory equipment:
- MiSeq instrument x2 (sequencing)
- Qiagen QiaCube x2 (extraction)
- Corbett Research Rotogene (Therascreen real-time PCR)
- Agilent BioAnalyzer
- BIO-RAD C1000 Thermal cycler (CFX96)

Supplementary Figure 1: Clinical implementation of an NGS-based diagnostic framework. **(A)** Workflow for the NGS-based mutational screening of treatment predictive alterations in lung cancer. Key turnaround time (TAT) metrics are displayed. **(B)** GANNT scheme of the workflow for the NGS mutational assay, with two parallel analyses running each week. Inclusion of a NanoString RNA based gene fusion assay is added to the NGS GANNT scheme, together with information about human resources, cost and larger laboratory equipment needed for the implementation. Reports from the NanoString assay can be issued at two different time points depending on whether a combined or separate reports are desired. **(C)** TAT for the molecular NGS part in calendar days for lung cancers analyzed during 2015 in the NGS central laboratory. **(D)** Total TAT including both the preanalytic and molecular laboratory time in calendar days for lung cancers analyzed during 2015.

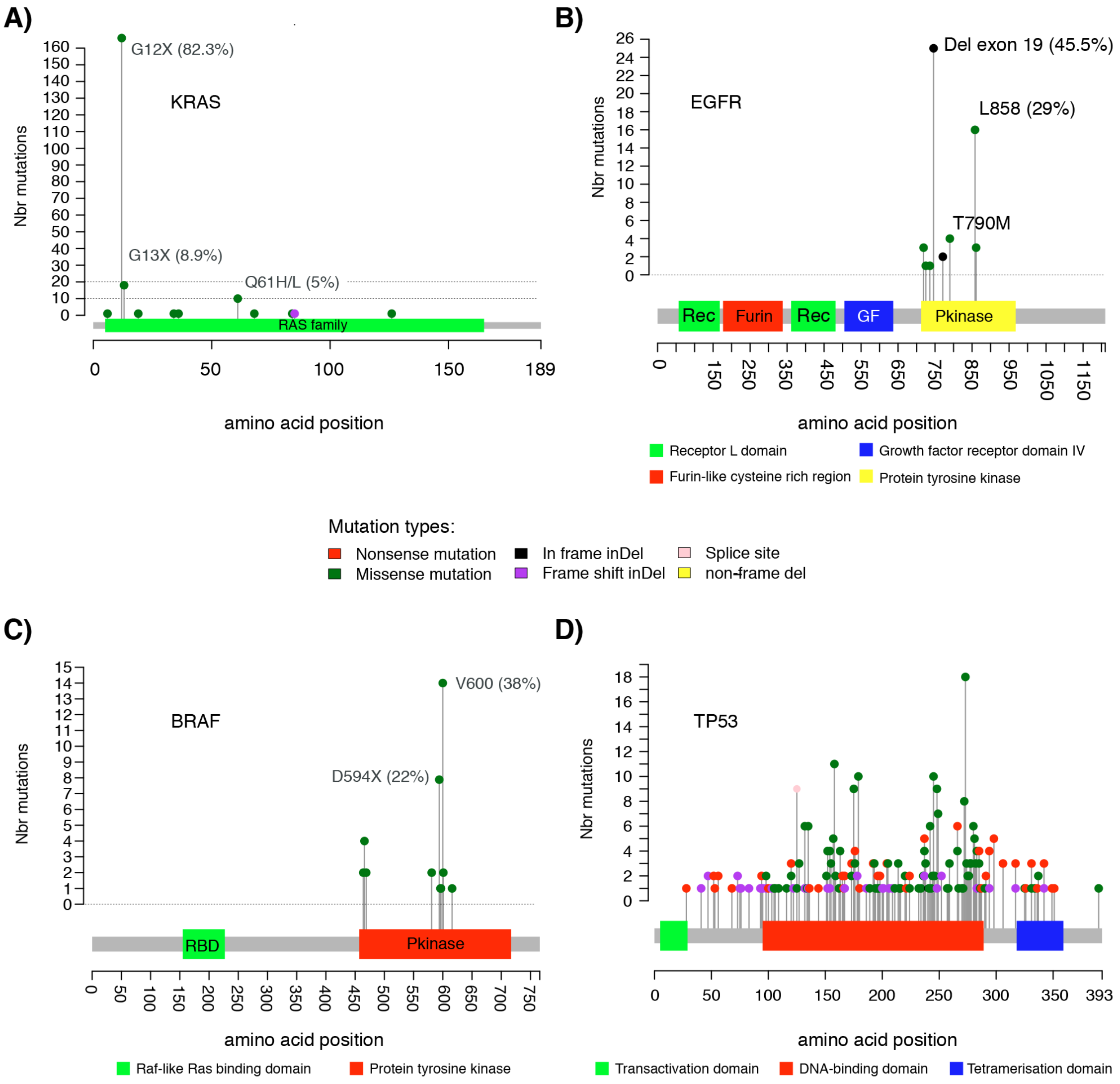

Supplementary Figure 2: Localization of detected mutations in *KRAS*, *EGFR*, *BRAF* and *TP53* in 533 consecutive NSCLC cases. **(A)** Protein localization of 203 *KRAS* variants. **(B)** Protein localization of 55 detected *EGFR* variants **(C)** Protein localization of 37 *BRAF* variants. **(D)** Protein localization of 321 *TP53* variants. In A to D, mutations are depicted at their respective amino acid site. Splice site mutations are excluded if not a specific protein position was provided. The height of the stems represents the number of mutations, while the color of the points represent the mutation type. Active protein domains are depicted as colored boxes. If multiple gene mutations are observed within a sample all mutations are included in the panels, thus displayed proportions are relative to a combined mutation spectrum, not patient proportion spectrum.

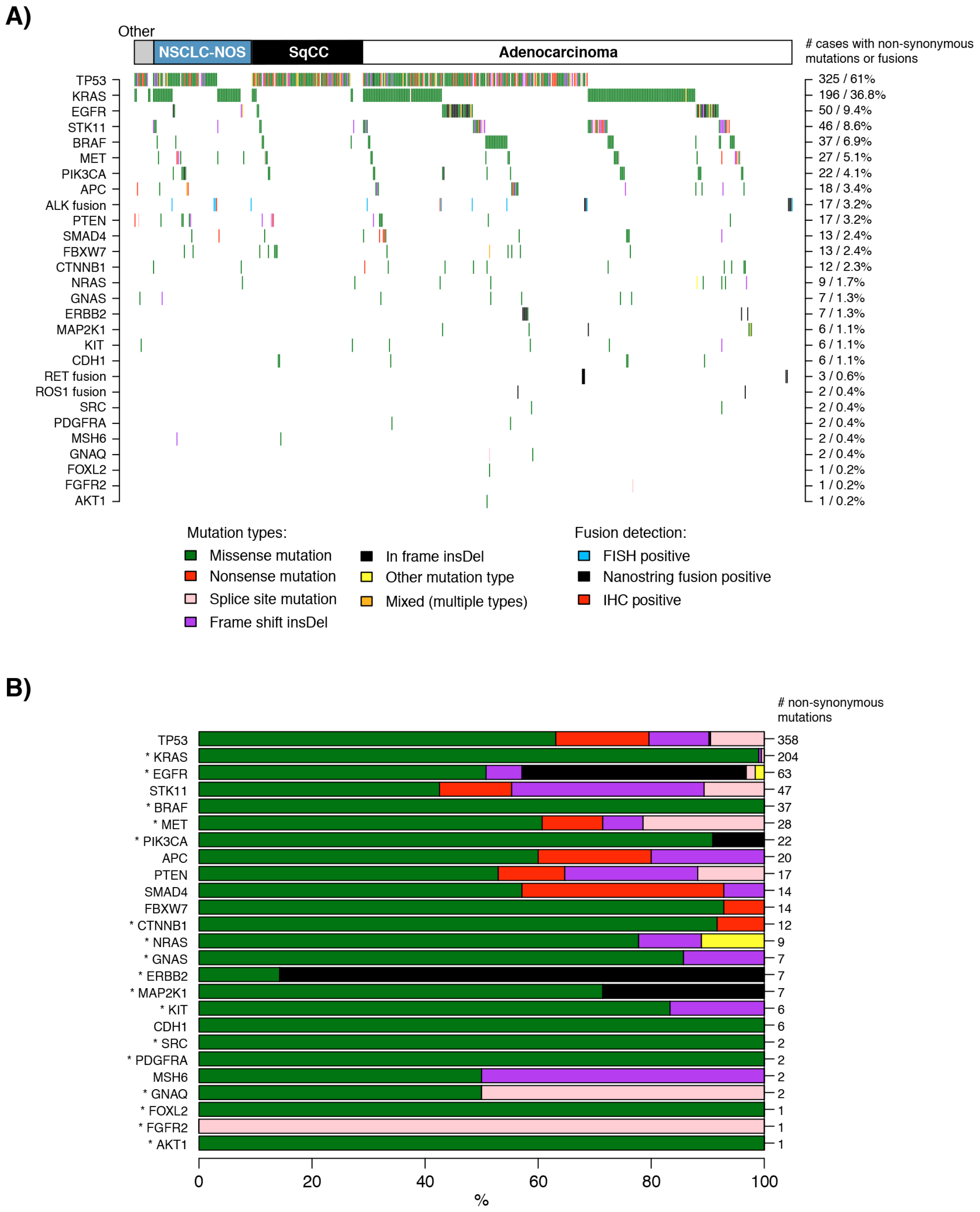

Supplementary Figure 3: Detected non-synonymous mutations and gene fusions in a consecutive 533-sample NSCLC cohort. **(A)** Heatmap describing gene fusions and non-synonymous variants and their mutation type in investigated genes identified in each case. Each column represents a sample; each row represents a gene. Numbers and proportions displayed on the right axis correspond to the total cohort (533 samples). **(B)** Proportions (%) of different mutation types as defined in panel A for non-synonymous mutations detected in analyzed genes. Numbers displayed on the right axis correspond to the total number of variants detected per gene in 533-sample cohort (a sample may harbor >1 variant per gene). * indicates oncogene.

Supplementary Table 1: Concordance between NGS and existing clinical diagnostics on the validation cohort

		NGS							
		***KRAS* mut**	***KRAS* wt**	***NRAS* mut**	***NRAS* wt**	***BRAF* mut**	***BRAF* wt**	***EGFR* mut**	***EGFR* wt**
**Prior clinical diagnostics technique**	***KRAS* mut**	**9**	**1***						
	***KRAS* wt**	**1****	**2**						
	***NRAS* mut**			**1**	**0**				
	***NRAS* wt**			**0**	**2**				
	***BRAF* mut**					**10**	**0**		
	***BRAF* wt**					**1****	**16**		
	***EGFR* mut**							**8**	**0**
	***EGFR* wt**							**1****	**32**

* : Discordant case between prior clinical diagnostic method and NGS.
** : Detected variant not analyzed by the prior clinical method.
wt: acronym for no mutation detected at investigated loci.

Supplementary Table 2: Associations between mutations/gene fusions and clinicopathological variables

See Supplementary File 1
